# Temperature as a modulator of allosteric motions and crosstalk in mesophilic and thermophilic enzymes

**DOI:** 10.3389/fmolb.2023.1281062

**Published:** 2023-10-09

**Authors:** Alexa L. Knight, Vinnie Widjaja, George P. Lisi

**Affiliations:** Department of Molecular Biology, Cell Biology and Biochemistry, Brown University, Providence, RI, United States

**Keywords:** NMR, thermodynamics, kinetics, temperature, allostery, thermophiles

## Abstract

Mesophilic and thermophilic enzyme counterparts are often studied to understand how proteins function under harsh conditions. To function well outside of standard temperature ranges, thermophiles often tightly regulate their structural ensemble through intra-protein communication (via allostery) and altered interactions with ligands. It has also become apparent in recent years that the enhancement or diminution of allosteric crosstalk can be temperature-dependent and distinguish thermophilic enzymes from their mesophilic paralogs. Since most studies of allostery utilize chemical modifications from pH, mutations, or ligands, the impact of temperature on allosteric function is comparatively understudied. Here, we discuss the biophysical methods, as well as critical case studies, that dissect temperature-dependent function of mesophilic-thermophilic enzyme pairs and their allosteric regulation across a range of temperatures.

## Introduction

Allostery is a regulatory mechanism that spatially and functionally couples distinct regions of a protein, allowing multiple sites to communicate. The use of allosteric modulators, such as small molecules, peptides, or mutations can alter enzymatic activity or substrate binding affinity via changes in structure or dynamics of the protein. The idea that subtle conformational motions can affect the transmission of chemical information over small and large molecular distances has been refined over the past decade, as an increasing number of allosteric systems only partially conform to classical paradigms necessitating specific sequences of biomolecular events. Experiments paired with theoretical underpinnings have confirmed that allostery can be driven primarily through protein motions and that allosteric effectors do not necessarily activate a protein through conformational change (Fenton, 2008; Amaro, 2017; Kremer and Lyssiotis, 2022). Central to this observation is the existence of amino acid networks that relay chemical information to modulate the degree of coupling between active and allosteric sites. “Ensemble” allostery has been observed in both mesophilic and thermophilic enzymes, where the effect of temperature on conformational ensembles, protein motions, and molecular crosstalk have been investigated to understand enzyme function at physiological extremes.

**FIGURE 1:**
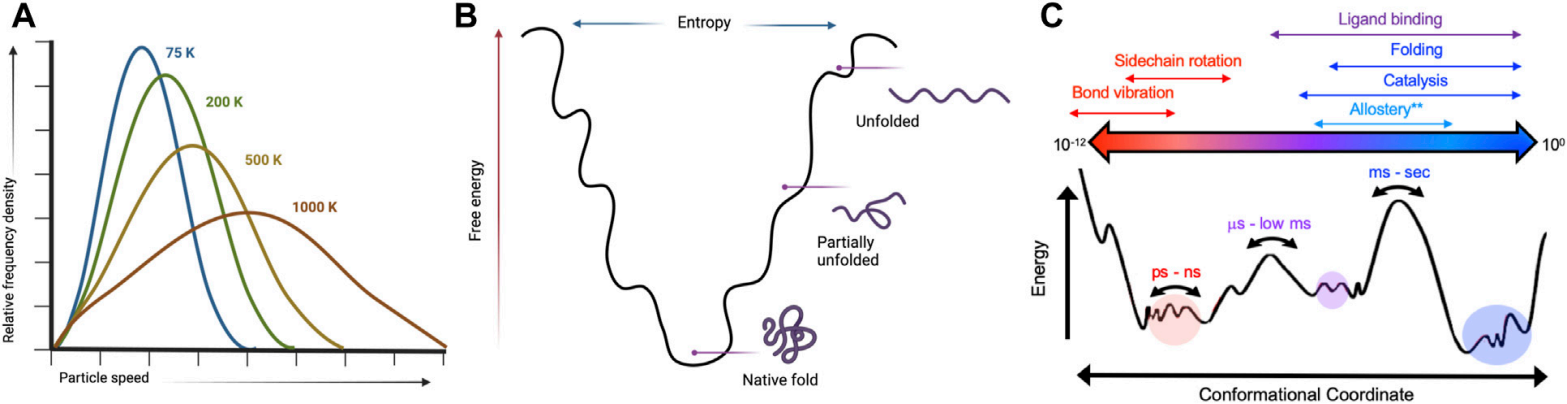
Kinetic and thermodynamic aspects of protein conformational equilibria are temperature-sensitive. **(A)** Temperature dependence of a Boltzmann distribution showing increased degeneracy at higher temperatures. Created with BioRender. **(B)** Protein folding landscapes rely on a delicate balance of enthalpy and entropy that are sensitive to temperature. Created with BioRender. **(C)** Many biological processes have distinct temporal and energetic requirements that define protein conformational states. Variable temperatures can skew energetic barriers that rewire protein motions to directly influence biochemical mechanisms.

The temperature dependence of a classical Boltzmann distribution describing kinetic energies for a set of molecules (Figure 1) can be extended to a description of protein conformational dynamics (Skrdla, 2009). Energy levels within such thermodynamic equilibria become increasingly degenerate as temperature increases, thereby allowing enzymes to escape kinetics barriers that drastically suppress active populations near room temperature. Allosteric communication and processes resulting from energetic connections between distal molecular sites correlate to the Boltzmann distribution of state transitions as temperature increases. Thus, temperature plays an important role in allosteric regulation, particularly in thermophilic enzymes that remain active over very wide environmental ranges (Braxton et al., 1994; McGresham et al., 2014; Lisi et al., 2018; Saavedra et al., 2018). For example, K-type enzymes (allosteric effector modulates *K*_D_) functioning at elevated temperatures have been found in some cases to exhibit an enhancement of allosteric function, while in others the allosteric effect is inverted (McGresham et al., 2014; McGresham and Reinhart, 2015). Temperature-induced allosteric modulation in most K-type enzymes, as expected, is driven by changes in substrate affinity upon allosteric ligand binding (McGresham and Reinhart, 2015; Katava et al., 2020; Maschietto et al., 2023). Beyond this effect, correlations between temperature elevation and enzymatic activity do not reveal consistent patterns regarding allosteric activation (Katava et al., 2017; Katava et al., 2020), thus, the molecular mechanisms underlying temperature-modulated allostery remain unclear. As a consequence, allosteric drug design against target proteins from extremophilic organisms (for example, opportunistic pathogens that survive processes like pasteurization) or for enzyme regulation under harsh conditions is understudied (Maschietto et al., 2023). In this review, we discuss the importance of temperature in distinguishing characteristics of allosteric enzymes and highlight case studies of enzymes that have temperature-dependent structures, dynamics, or functions as well as meso-thermophilic paralogs that maintain structure, but differ in key biophysical ways. We also highlight current biophysical methods for interrogating the influence of temperature on protein conformation and dynamics.

### Current tools for interrogating allosteric systems via temperature

#### Solution nuclear magnetic resonance (NMR) spectroscopy

Solution nuclear magnetic resonance (NMR) spectroscopy is a premier molecular tool to elucidate structural and dynamic changes in allosteric systems. NMR is highly sensitive to local environments (*i.e.*, subtle changes to protein structures) and can probe molecular motion on a wide range of biological timescales (picosecond-second). Of critical importance are the biologically slow motions that traditionally define conformational equilibria associated with protein folding, enzyme catalysis, and allosteric communication (Figure 1) (Cavanagh, 2007; Lisi and Loria, 2016). NMR experiments designed to quantify protein motion related to these processes are called relaxation dispersion (Neudecker et al., 2009; Palmer, 2016). These experiments provide kinetic, thermodynamic, and structural information as micro-millisecond protein motions broaden NMR resonances by increasing the transverse relaxation rate (*R*_2_) by an amount *R*_ex_. In the fast exchange limit, *R*_ex_ can be described as

$$R_{ex}=\frac{P_{A}P_{B}{∆\omega }^{2}}{k_{ex}}$$ (1)

where k_ex_ is the chemical exchange rate between the assumed two conformational states (kinetics), *P*_A_ and *P*_B_ are the equilibrium populations of the conformers (thermodynamics), and Δω is the chemical shift difference between conformers (structure). The use of Eq. 1 in the mathematical expressions for single- and multiple-quantum Carr-Purcell-Meiboom-Gill (CPMG) and *R*_1_ρ relaxation experiments has been derived elsewhere (Cavanagh, 2007; Lisi and Loria, 2016; Neudecker et al., 2009; Palmer, 2016).

Solution NMR can be performed under a variety of physiological pH or solvent conditions (Lisi and Loria, 2016), but of particular interest here is the effect of temperature on the parameters described above. Indeed, some earlier NMR studies have outlined structural and dynamic alternations in proteins across wide temperature ranges (Morino et al., 1984; Ju et al., 2004; Weininger et al., 2014), while others have focused on utilizing temperature variations to probe the underlying causes of evolutionary divergence in pairs of thermophilic and mesophilic enzymes (Butterwick et al., 2004; Wolf-Watz et al., 2004; Toth et al., 2009). In the latter case, it is suggested that temperature-dependent allosteric regulation (i.e., where allosteric effects “accelerate” or “dampen” with temperature) may be an evolutionary feature to maintain maximal catalytic efficiency of an enzyme over a wide range. Enzymes classified as either mesophilic or thermophilic often display similar dynamic signatures under “traditional” regimes of 273–290 K. Conformational fluctuations that distinguish these enzyme systems from one another become more apparent as the temperature approaches substantially higher (or lower) regimes. This can manifest as a change in k_ex_, structure (Δω), or population of active conformer at a given temperature. The relationship between k_ex_ and Δω influences how the NMR resonances appear and may be manipulated by temperature to provide a quantitative link between protein motion and activation (via ΔG^≠^, which is related to *E*_*a*_) (Ruminski et al., 1998; Westwood et al., 2022). Scenarios where k_ex_ is much greater than, equal to, or much less than Δω dictate the fast, intermediate, or slow exchange regimes, respectively. At the extremes of this continuum, an NMR resonance appears as a population-weighted average (fast exchange) or as two resonances with populations defined by the peak volumes (slow exchange).

The reshuffling of structural populations is critical to the activation of enzymes as allosteric modulators bias the ensemble toward an active state. Enzyme activation pathways can therefore be observed in real-time by NMR, and changes in sample temperature can alter the way enzymes achieve their functional conformation. In the fast exchange limit, where k_ex_ >> Δω and NMR resonances appear as a population-weighted average, the single N-H chemical shift contains information about the major and minor states.

$$\delta_{NH}=p_{b}\omega_{b}+p_{a}\omega_{a}$$ (2)

where Δω is defined as |ω_b_ − ω_a_|, the characteristic chemical shifts of each conformer. The relationship between populations of exchanging states can define *K* (Bain et al., 1995; Krishnan, 2019), the equilibrium constant related to Δ*G* by

$$∆G=-RTln\left(\frac{p_{b}}{p_{a}}\right)$$ (3)

The transition state energy for the conversion between states (*i.e.*, change in equilibrium populations) can be estimated from the temperature dependence of the NMR linewidth (Δν_1/2_), fitting to Eq. 4.

$$∆\upsilon_{1/2}=\frac{1}{\pi T_{2}}+\frac{K{\left(\omega_{b}-\omega_{a}\right)}^{2}}{4\pi \left(1+K\right)}\left(\frac{h}{k_{B}T}\right)e^{{∆G}^{\neq }/RT}$$ (4)

Here, *K* is defined by the ratio of *P*_B_ and *P*_A_. Calculation of ΔG^≠^ provides access to the individual components of k_ex_ (k_ex_ = k_f_ + k_r_) through the Eyring Equation (Ruminski et al., 1998).

$$k_{f}=\kappa \frac{k_{B}T}{h}e^{{-∆G}^{\neq }/RT}$$ (5)

where κ is assumed to be equal to 1. Analysis of k_f_ and k_r_ can dissect aspects of k_ex_ that are commensurate with catalytic rates, open-to-close motions of activation loops, or enzyme breathing. Indeed, thermodynamic contributions to the transition state between inactive and active enzyme can be estimated from the linear form of the Eyring equation

$$\ln\frac{k_{f}}{T}=\frac{{∆H}^{\neq }}{R}\frac{1}{T}+\ln\frac{\kappa_{B}}{h}+\frac{{∆S}^{\neq }}{R}$$ (6)

where transition state enthalpy and entropy are the slope and *y*-intercept of the plot, respectively. The Arrhenius equation (*k* = *A*e^−Ea/RT^) describing the effect of temperature on reaction rates via transition state energy is similar and can be considered if *k*_ex_ is commensurate with reaction rate (Carvalho-Silva et al., 2019). Here, the pre-exponential factor, *A*, is slightly temperature-dependent, rather than constant and reaction rates with large *E*_a_ increase rapidly with temperature. Some similarity between the Eyring and Arrhenius relationships requires an assumption of constant enthalpies and entropies of activation, nonetheless, reaction rates altered by the presence of allosteric ligands may be highly temperature-dependent, suggesting that NMR studies at multiple temperatures may be required to define a mechanism, particularly for enzymes derived from extremophilic organisms (Siemer et al., 2012; Holliday et al., 2017; Busi et al., 2021).

As allosteric effectors shift the structural ensemble of an enzyme, the weak interactions surrounding the enzyme active site may also be altered substantially. Subtle bond vibrations and side chain reorientations that are essential to the active states of most enzymes break and/or form van der Waal’s contacts and hydrogen bonds. NMR has been successful in estimating hydrogen bond lengths and propensities via temperature-dependent ^1^H-^15^N chemical shifts. Straightforward HSQC measurements over a wide temperature range provide Δδ_HN_/ΔT, where values below a certain threshold (often defined as −4.6 ppb/K in the literature) indicate a significant hydrogen bond propensity for a given residue (Cierpicki and Otlewski, 2001). The magnitude of Δδ_HN_/ΔT has been correlated to the hydrogen bond length, and evaluation of temperature-dependent ^1^H-^15^N chemical shifts across a series of point-mutants, or between meso- and thermophilic enzymes of the same class, can provide fairly detailed structural information to complement relaxation dispersion studies (Belato et al., 2022).

#### Isothermal titration and differential scanning calorimetry

Thermodynamic comparisons of meso- and thermophilic proteins can provide the framework for understanding functional differences related to allostery. Isothermal titration calorimetry (ITC) is a thermodynamic technique that determines changes in enthalpy (*ΔH*), entropy (*ΔS*) and free energy (*ΔG*) that is readily applied to complex systems where ligands bind cooperatively at multiple sites involving protonation/deprotonation events, allosteric effectors, changes in oligomeric states, or conformational changes (Eftink et al., 1983; Freiburger et al., 2009; Ghai et al., 2012). Although ITC is sensitive to the energetics of conformational transitions and macromolecular interactions, the temperature dependence of the ligand binding parameters is rarely measured, challenging the quantitation of complex binding equilibria by ITC alone. This is especially true for mesophilic proteins with thermophilic counterparts that often differ in ligand binding thermodynamics despite similar structures. Another challenge of some calorimetric analyses is the assumption that the change in heat capacity (*C*_p_) upon effector binding [or protein (un) folding] remains constant or is unaffected by temperature. While dominated by solvent due to the high heat capacity of water, Δ*C*_p_ can be experimentally calculated from slopes of plots of ITC-measured *ΔH* as a function of temperature and incorporated into van’t Hoff analyses of thermodynamics describing allosteric processes. In several studies, thermophilic proteins have been found to be substantially more rigid at room temperature (insight extracted partially from Δ*C*_p_) with lower than expected temperatures of binding and Δ*C*_p_ (Datta and LiCata, 2003).

Differential scanning calorimetry (DSC) is a similarly powerful technique used to characterize temperature-induced changes in protein stability, *i.e.*, the difference between the free energies of the native and the unfolded states (Δ*G*_unf_ = *G*_U_ − *G*_N_). The temperature at which the Δ*G*_unf_ is equal to zero defines the thermal denaturation midpoint (*T*_m_), and the dependence of protein stability on temperature is also influenced by Δ*C*_p_, which can be measured empirically by the difference in pre- and post-transition baselines of the DSC unfolding profile. The magnitude of Δ*C*_p_ is typically positive in DSC measurements, due to the liberation of solvent during unfolding, while effector binding studies via ITC often yield negative Δ*C*_p_ due to desolvation. DSC studies of thermophilic systems, like those of ITC, commonly display lower Δ*C*_p_ values that contribute to higher measured *T*_m_. Energetic differences in meso- and thermophilic species can be readily determined with DSC, as can the respective contributions of specific amino acids to overall structural stability or allosteric crosstalk. A “reference temperature” is required to extrapolate the thermodynamic parameters determined at *T*_m_ (where Δ*G* is equal to zero) for a van’t Hoff analyses, where Δ*H* and Δ*S*, at the reference temperature (*T*_ref_) can also be calculated.

$$∆H\left(T_{ref}\right)=∆H\left(T_{m}\right)+\int_{T_{m}}^{T_{ref}}{∆C}_{p}dt$$ (7)

$$∆H\left(T_{ref}\right)=∆H\left(T_{m}\right)+{∆C}_{p}\left(T-T_{m}\right)$$ (8)

where Δ*H*(*T*_m_) results from the direct integration of the area under the calorimetric curve. Often, Δ*C*p can be determined from the difference in the pre- and post-unfolding transition baselines, or calculated based on an average of ∼14 cal/molK for each amino acid (Privalov et al., 1995; Privalov and Dragan, 2007). Using the same direct calorimetric measurements, Δ*S* can be extrapolated to a reference temperature by Eqs 9, 10.

$$∆S\left(T_{ref}\right)=\frac{∆H\left(T_{m}\right)}{T_{m}}+\int_{T_{m}}^{T_{ref}}{∆C}_{p}\ln\;dt$$ (9)

$$∆S\left(T_{ref}\right)=\frac{∆H\left(T_{m}\right)}{T_{m}}+{∆C}_{p}\;\ln\left(\frac{T_{ref}}{T_{m}}\right)$$ (10)

Such an analysis assumes Δ*C*_p_ is a temperature-independent parameter, though earlier discussion of kinetic and thermodynamic principles via Eyring and Arrhenius show this is not the case. The resulting free energy change at a given temperature is then calculated using classical thermodynamic relationships, or determined via measured Δ*H*(*T*_m_) and Δ*Cp* values.

$$∆G\left(T_{ref}\right)=∆H\left(T_{m}\right)\frac{T_{m}-T_{ref}}{T_{m}}+\int_{T_{m}}^{T_{ref}}{∆C}_{p}\;dT-T_{ref}\int_{T_{m}}^{T_{ref}}{∆C}_{p}dlnT$$ (11)

$$∆G\left(T_{ref}\right)=∆H\left(T_{m}\right)\left(1-\frac{T_{ref}}{T_{m}}\right)-∆C_{p}\left[T_{m}-T_{ref}+T_{ref}\ln\left(\frac{T_{ref}}{T_{m}}\right)\right]$$ (12)

Though calorimetric measurements provide valuable information about the energetics of binding, conformational changes, or structural stability, these methods do not provide the high-resolution detail about specific aspects of structure or mechanism that are influenced by allosteric modulators or that distinguish mesophilic and thermophilic species of the same enzyme. Thus, an integrated approach of several methods is optimal for detailed mechanistic studies (Monod et al., 1965; Popovych et al., 2006; Diehl et al., 2010; Hobbs et al., 2013).

#### Temperature dependent X-ray crystallography

At room temperature, biological macromolecules remain in motion in the crystalline state, where the macromolecular crowding and solvent content of the crystal are similar to those inside a cell. At cryogenic temperatures, macromolecular motions are slowed or halted, impairing biological activity. Thus, the temperature at which a crystal is stored (100 ≥ T ≤ 273 K) can act as an “on/off” switch that tunes protein motion. Exploiting temperature-controlled X-ray crystallography to capture proteins in action is therefore essential to providing the atomic level snapshots that most accurately resemble the conformational ensembles measured in solution, including sparsely-populated intermediates that are otherwise invisible, which in allosteric systems can represent “pseudo-active” states (Weik and Colletier, 2010). Capturing such states by sampling at variable temperature can elucidate the vastness of conformational changes in a given system (Du et al., 2023).

**FIGURE 2:**
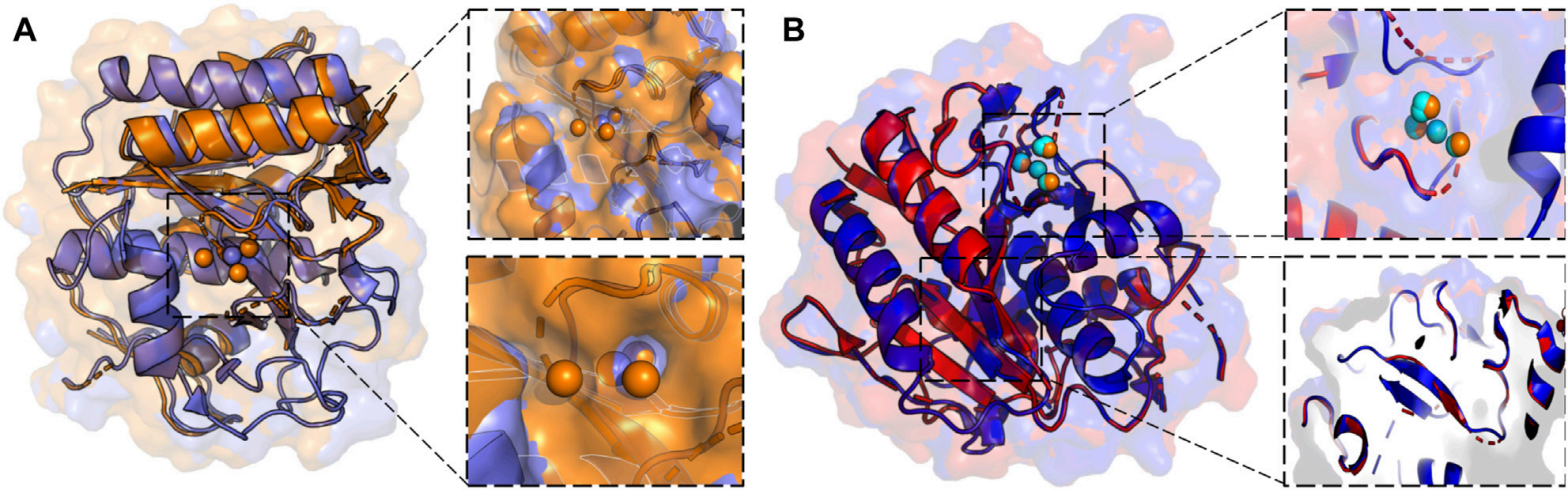
**(A)** X-ray crystallographic overlay of Proteinase K structures collected at 100 K (PDB ID:7LTD, blue) and 363 K (PDB ID:8SOU, orange) highlighting temperature-dependent differences in the backbone conformation and ligand binding pocket. A Ca^2+^ binding pocket in Proteinase K, showing four distinct binding locations at 363 K, but only a single binding mode at 100 K. **(B)** X-ray crystallographic overlay of Proteinase K structures collected at 313 K (PDB ID: 8SOG, red) and 363 K (PDB ID: 8SOU, blue) showing differences the backbone and side chain conformation, as well as ligand binding pocket. Ca^2+^ binding locations once again vary at two elevated temperatures and are represented in cyan at 313 K and orange at 363 K.

X-ray crystallography leverages cryogenic temperatures (∼100 K) to avoid radiation damage or degradation of the sample while improving diffraction data quality (Dou et al., 2020). However, recent advances in room temperature detection, sources, and optics have facilitated explorations of conformational heterogeneity and by extension, the inference of related molecular function. Degrees of freedom present at room temperature are suppressed during cryo-cooling, altering the conformational landscape. Additionally, at temperatures of ∼180–200 K, proteins can undergo glass transitions, leading to cryo-artefacts that cloud the interpretation of low-temperature crystallographic structural features (Dou et al., 2020). More recent advances using Laue diffraction at synchrotron radiation sources have led to the development of time-resolved X-ray crystallography, *i.e.*, the collection of “dynamic data” using a continuous stream of microcrystals that are irradiated for only a few femtoseconds. Time resolution at room temperature enables the rapid motions of proteins and visualization of conformational changes during chemical reactions (Brändén and Neutze, 2021). Such an advance positions time-resolved X-ray crystallography as an emerging method for the observation of multi-temperature chemical reactions and conformational states. Current approaches to collecting X-ray diffraction data above room temperature must circumvent radiation damage by limiting initial testing exposures, reducing X-ray dosage and exposure time, or utilizing novel materials as sample holders (Roedig et al., 2015; Dou et al., 2020). Such strategies were leveraged in the study of proteinase K by Fraser and coworkers, enabling X-ray diffraction to be collected at 313K, 333K, 343K, 353K, and 363 K. The resulting models of alternative backbone and side chain conformations, as well as extensive and disparate conformational changes across the temperature range (including the elucidation of previously invisible ligand binding site, Figure 2), illustrates the power of time and temperature resolution in X-ray crystallography (Du et al., 2023).

#### Molecular dynamics simulations

Molecular dynamics (MD) has been widely adapted to studies of allosteric mechanisms due to its natural complementarity to NMR and single-molecule spectroscopies. MD offers a glimpse into the energy landscape of proteins, and therefore insight into the balancing act between ordered catalytic sites and dynamic disorder of the surrounding protein matrix. MD has allowed for the cost-efficient sampling, identification, and ranking of temperature sensitive contacts critical for thermally stable behavior and been instrumental in the quantitation of free energies of cooperativity that account for changes in the amplitudes of protein vibrational motions the describe the conformational ensemble (McClendon et al., 2009; East et al., 2020). MD has effectively described allosteric effects analytically through vibrations around a static structure via coarse-grained (Pak and Voth, 2018), normal-mode analysis (Bauer et al., 2019), and all-atom ensembles through concomitant analysis of cross-correlations (Brown et al., 2017), contact correlations, principal components or local unfolding correlations (McClendon et al., 2009). Several metrics have been developed to quantitate correlated motions from MD simulations. For example, backbone and sidechain motions monitored through internal coordinates have been shown to account for “gear-like” twists that occur in the absence of the large conformational changes observed in classical allosteric proteins (McClendon et al., 2009). Other strategies include mutual information metrics from many short simulations to elucidate stepwise mechanisms of cooperative small molecule binding. As this approach identifies correlated conformations in an unbiased manner, it has emerged as a useful tool for the discovery of novel or “orphan” allosteric sites in proteins (McClendon et al., 2009).

MD has the capability to address questions regarding the mechanisms of related enzymes with identical structure, but variable thermal and kinetic behaviors. Specifically, in the case of mesophilic and thermophilic pairs, MD can reveal the presence of temperature-sensitive residues responsible for activity tuning (Burns et al., 2022). Simulations across a large temperature range, particularly those employing enhanced-sampling schemes can systematically modulate the strength of nonbonded interactions between atoms, which affects conformational sampling. Enhanced-sampling MD trajectories produce highly accurate melting profiles and folding temperatures and can be used to calculate inter-residue contacts across all temperatures. Contact matrices describing the percentage of simulation time for which any given residue pair interacts allow for the perturbation of these contacts to be assessed, for example, in thermophilic enzymes that are structurally or functionally sensitive to specific temperature regimes. The mutation of the temperature sensitive contacts, either *in vitro* or *in silico*, can further dissect the chemical importance of each residue to protein function at any given temperature, allowing the results of temperature-dependent MD to iteratively tune an enzyme to return the highest possible activity.

### The effect of temperature on allosteric communication

Allosteric mechanisms, essential for the transmission of perturbations from an effector binding site to other distant functional sites, are widespread and often depend on protein conformational fluctuations (Lange et al., 2008). In fact, biomolecular motions involved in conformational selection provided some of the early insight into dynamically driven allostery that did not conform to classical paradigms (Lange et al., 2008). The extent and timescale of protein motion is intimately linked to ligand affinity or the degree of enzyme activation via the effective sampling of productive conformational states. The temperature-dependent conformational or kinetic equilibria that define allosteric mechanisms can be tied to entropy (Diehl et al., 2010; Caro et al., 2017; Wand and Sharp, 2018), change in heat capacity (Hobbs et al., 2013), or enthalpy (Monod et al., 1965), but many elegant studies have demonstrated that the nature of this long-range coupling is not universal, and the highly varied thermal properties between and within enzyme families can strongly affect allostery. The latter observation prompts questions as to whether allostery is even the primary mechanism at the physiological temperature of many enzymes, particularly thermophiles that are often studied at lower, non-native temperatures. Thus, the temperature-dependence of allostery requires a deeper consideration, particularly for thermophilic enzymes with wide activity ranges. Studies of the adaptability to temperature in several enzymes has shown that enthalpic, and more critically, entropic tuning is a viable method to modulate the energetic favorability of allosteric pathways, which will be discussed in the case studies below.

#### Case study: temperature-dependent allosteric activation of a thermophilic enzyme

Biophysical studies of thermophilic enzymes offer unique insights into the molecular factors that distinguish them from mesophilic paralogs, which is a decades old question of protein biochemistry. Thermophiles are often easier to work with than corresponding mesophiles, and in this case study, provide an opportunity to scrutinize temperature-sensitive aspects of allostery. Loria and coworkers have shown in several studies that the glutamine amidotransferase imidazole glycerol phosphate synthase (IGPS) is a model for V-type allostery (allosteric effector impacts catalytic *V*_max_) that can be structurally and enzymatically characterized over a very wide temperature range (Lisi et al., 2017; Lisi et al., 2018). *Thermatoga maritima* IGPS is a heterodimeric complex of the HisH (glutaminase) and HisF (cyclase) enzymes, where the two catalytic sites are separated by >25 Å yet are functionally synchronized. HisF binds the allosteric activator N′-[(5′-phosphoribulosyl)formimino]−5- aminoimidazole-4-carboxamide-ribonucleotide (PRFAR), which is entropically driven at room temperature, and enhances glutamine hydrolysis in HisH nearly 5000-fold over basal levels.

**FIGURE 3:**
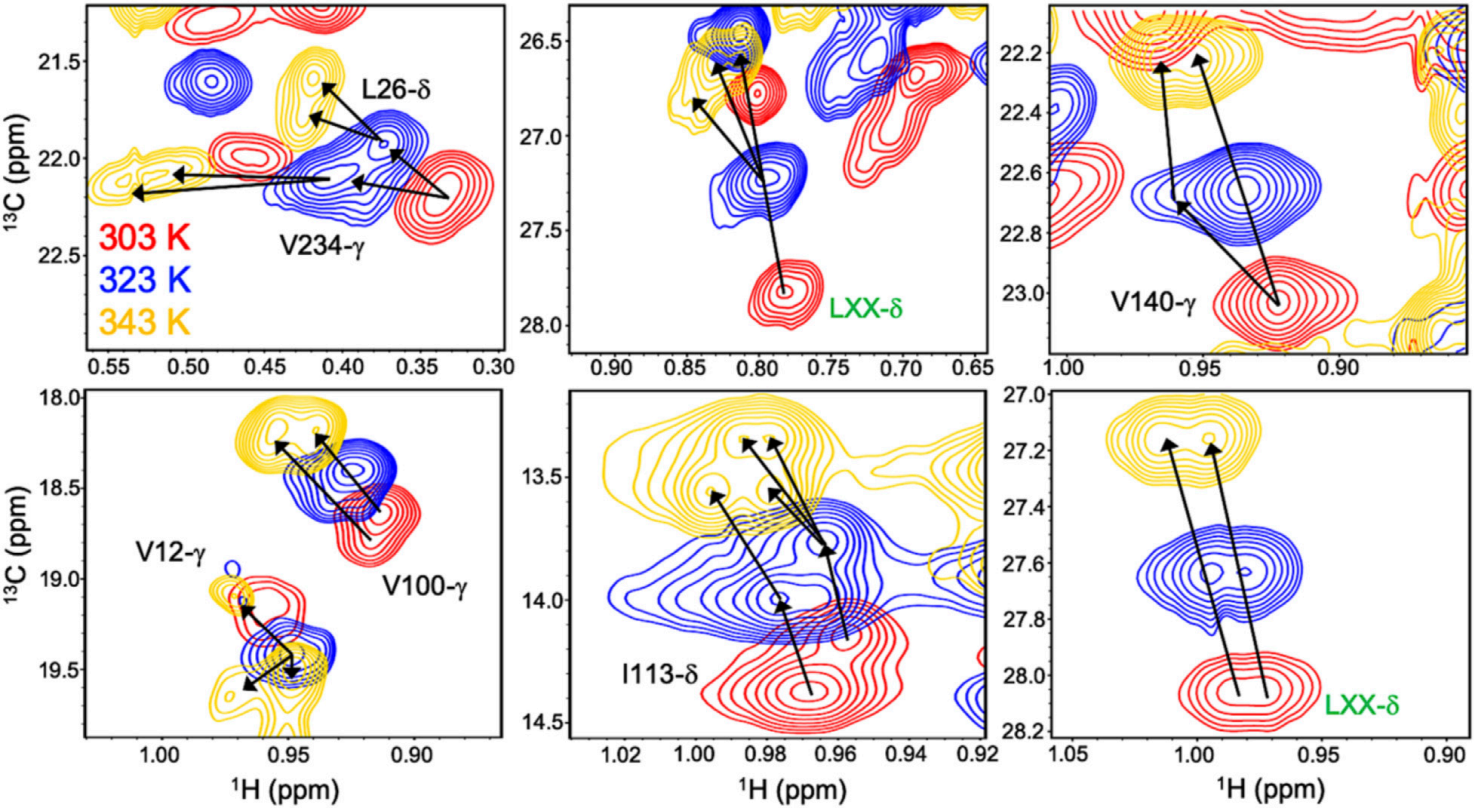
Overlay of ^1^H-^13^CH_3_ methyl-ILV NMR spectra collected at 303 K (red), 323 K (blue), and 343 K (gold) depicting temperature-dependent variations in exchange regime. Arrows following chemical shift trajectories bifurcate when minor populations of a given resonance become visible. Here, elevated temperatures more clearly resolve multiple conformations within the HisF subunit of IGPS. Resonances labeled in green were unassigned in the original data presented by Loria and coworkers in *Front. Mol. Biosci.* **2017**.

Allosteric activation of Gln hydrolysis by PRFAR is driven by milisecond (ms) timescale motions throughout the HisF domain that propagate to the dimer interface and reposition the glutaminase active site architecture for catalysis. Mutations along the “dynamic pathway” spanning HisF and HisH diminished ms motion and catalytic rates to a similar extent (Lisi et al., 2017). The link between PRFAR-induced allosteric activation and protein dynamics was established at 303 K, well below the physiological temperature of *T. maritima*. Temperature-dependent NMR studies of ^1^H^13^CH_3_ ILV-methyl labeled apo IGPS over a range of 303–343 K revealed linear, temperature-dependent shifts in fast exchange (k_ex_ >> Δω) (Lisi et al., 2018). At 343 K, surprisingly, a cluster of methyl resonances appeared to experience slow exchange (Figure 3). PRFAR-bound IGPS displayed similar behavior based on ^1^H-^13^C temperature coefficients, though slow exchange at 343 K was redistributed to different residues. Sites found to undergo ms motion in response to PRFAR binding were notable outliers when comparing apo- and PRFAR-bound IGPS temperature coefficients, suggesting that IGPS retains its flexibility at high temperatures.

**FIGURE 4:**
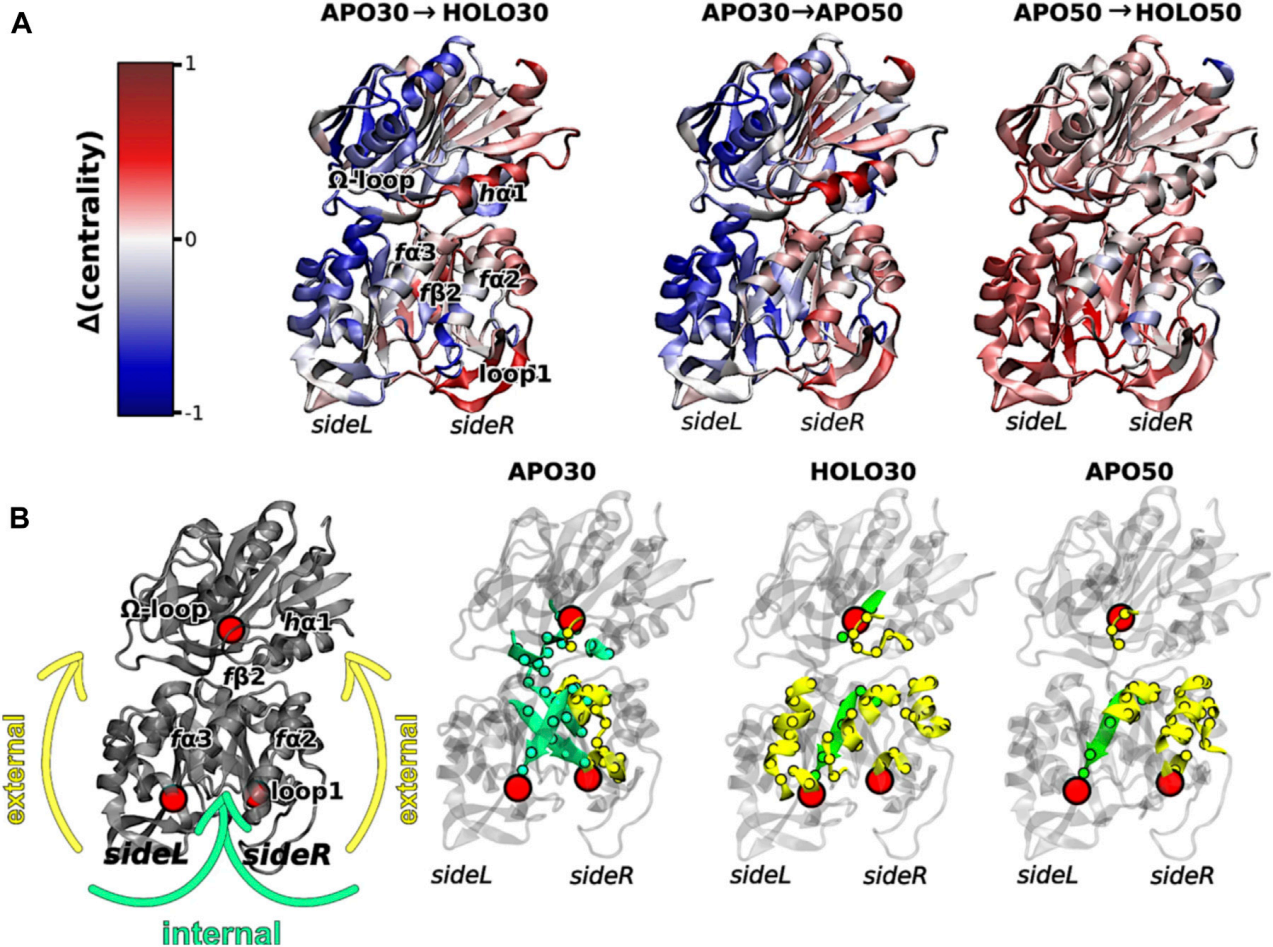
**(A)** Differences in Eigenvector Centrality within IGPS, intrinsic to the binding of PRFAR (holo enzyme) at 303 K (left) or 323 K (right) and without effector binding (apo enzyme) at the elevated temperature of 323 K (center). Communication pathways induced by PRFAR at 303 K are recapitulated solely due to the effect of temperature in the apo enzyme at 323 K. **(B)** Comparative transmission of distinct mutual information pathways linking the PRFAR binding site to the active site (*h*G50 in HisH, red sphere). Areas of secondary structure involved in communication pathways are separately highlighted as solvent exposed amino acids (yellow, “external”) and buried (green, “internal”). Similar trends are observed in for the holo enzyme at 303 K and apo enzyme at 323 K when compared to apo IGPS (303 K), specifically a transition from internal pathways to external communication due to effector binding and/or temperature increase. Figure was reproduced from Maschietto et al. (2023), *Nature Communications*, under a Creative Commons Attribution 4.0 International License.

Interestingly, elevated temperatures enhanced the flexibility of apo IGPS substantially, so much so that the apo enzyme appeared dynamically similar to PRFAR-bound IGPS at 343 K, based on methyl-TROSY relaxation dispersion. Concomitant with this biophysical change, the degree of catalytic rate enhancement afforded by PRFAR at 343 K is only 65-fold (compared with ∼5000-fold at 303 K), despite a largely temperature-independent PRFAR binding affinity. Thus, at temperatures more closely approximating the native environment of *T. maritima*, IGPS appears to rely much less heavily on dynamic allosteric activation. To understand the molecular basis for this effect, MD simulations at elevated temperatures showed that temperature-driven increases in Eigenvector Centrality (EC), an established metric to assess dynamic connectivity along an allosteric pathway (Maschietto et al., 2023), mimicked those induced by PRFAR at room temperature. This suggested that temperature alone can compensate for the effect of PRFAR in establishing the allosterically activated IGPS conformation. When PRFAR binding was examined with EC at elevated temperatures, the crosstalk within the IGPS heterodimer encompassed the entire structure, consistent with biochemical data indicating a shift from well-defined allosteric rate enhancement to a “pre-activated” system with a highly flexible apo state (Figure 4).

#### Case study: temperature adaptation of dynamic allostery

Thermal adaptation of organisms to their environmental niches is a hallmark of evolution. When two descendants evolve under different temperature extremes, physicochemical differences (*i.e.*, adaptive mutations) arise that change how those enzymes catalyze essential reactions. Primary sequence variations are often found in residues that are surface exposed, while active sites in the protein interior are rarely structurally perturbed, preserving overall catalytic rates. An open question exists regarding how related enzymes with distinct thermally adapted sequences use allosteric changes to propagate information to/from the protein periphery to the active site. One explanation for this phenomenon may be “entropy-tuning,” a dynamic allosteric mechanism demonstrated by Hilser and coworkers through engineering of *Escherichia coli* adenylate kinase (Adk). Here, the conformational entropy of several distal regions serves as a metric for dynamically tuned ligand affinity and catalysis (Schrank et al., 2013). The three domains of Adk, the CORE, AMP-binding, and LID, work in concert to catalyze effective phosphoryl transfer. The highly stable CORE positions catalytic residues to form the substrate binding site, while the flexible LID and AMP-binding domains are spatially locked during binding and until product release. It was initially assumed that conformational changes in the LID and AMP-binding domains were rigid-body motions, but more recent work revealed a “local unfolding” of the LID domain via uncorrelated motions throughout the entire subunit.

Differential scanning calorimetry (DSC) studies of single-point mutations within the LID domain, created to increase the probability of local unfolding without disrupting the ground state structure, showed a clear reduction in the major unfolding transition of wild-type (wt) Adk. A smaller low-temperature transition in LID mutants corresponding to an increased population (∼40%) of a locally unfolded intermediate state was also observed, which comprises only 5% of wt-Adk at 305 K. The thermodynamic modulation of the minor LID conformer was recapitulated in temperature-dependent NMR relaxation experiments of apo- and holo-Adk LID variants. The stabilization of a locally unfolded minor state was attributed to the entropic favorability of removing bulky side chains that would normally restrict partial unfolding. Lower NMR sample temperatures return the LID conformational equilibrium to a wt-Adk level, illustrating quite elegantly that strategies for temperature adaptation can leverage the energetic (*i.e.*, entropic) balance of local unfolding, as only single-point mutations on the protein surface are sufficient to energetically bias the stability of the folded state of a domain within an allosteric complex. Olsson and Wolf-Watz provided a similar example using point mutations to partially unfold the ATP binding subdomain that would otherwise remain folded, revealing a novel open-to-close (or unfold-to-refold) molecular mechanism in *E. coli* Adk (Olsson and Wolf-Watz, 2010)*.* Additionally, as chaperone machinery has evolved to recognize unfolded states of proteins, local unfolding is an attractive mechanism for the regulation of enzymes via *K*_m_ or k_cat_ that are sensitive to a folded-unfolded equilibrium. Additional biophysical characterizations of local disorder in canonical enzymes are required to tie protein-level regulation to that of proteome-level regulation via chaperones.

#### Case study: engineering meso-thermophilic hybrid enzymes for thermostability and meaningful low-temperature activity

In numerous biotechnological applications, protein thermal stability is critical, as activity at higher temperatures can improve bioreactor capacity (Asial et al., 2013). There remains significant interest in improving the thermal properties of mesophilic enzymes via rational design and directed evolution, which now incorporates structure-based recombination, where stable folds such as the TIM barrel have been utilized to generate new “thermophilic” hybrid constructs. Several groups have hybridized mesophilic and thermophilic protein structures as a strategy for modulating protein allostery and the functional range of enzymes traditionally optimized for low-temperature activity (Burns et al., 2022). A recent example of such work leveraged mesophilic and thermophilic variants of the C-terminal domain of a bacterial Enzyme I (called eEIC and tEIC respectively) to produce hybrid EICs with modular thermal stability, active site flexibility, and allosteric function.

EI is an essential component of the phosphoenolpyruvate (PEP)-dependent sugar phosphotransferase system (PTS) pathway and a regulator of bacterial metabolism, making it an attractive drug target. Structurally, EI is a heterodimer connected by an unstructured linker, where the N-terminal domain supports the catalytic phosphoryl-transfer reaction, while the C-terminal domain governs EI dimerization and allosteric activation of PEP for catalysis (Dotas et al., 2020). Mesophilic (*E. coli*, eEIC) and thermophilic (*Thermatoga tengcongensis*, tEIC) constructs of EI have fully conserved active sites, maintain optimal activity at temperatures of 37 °C and 65 °C, respectively, and rely on conformational dynamics for effective interdomain crosstalk and catalysis (Dotas et al., 2020). However, the enzyme pair has only ∼50% overall sequence conservation. Rather than a single catalytically active state, Venditti and coworkers showed EIC to adopt multiple catalytic conformations, though eEIC and tEIC favor different conformational ensembles. Mesophilic eEIC samples its active state more frequently, suggesting that structural heterogeneity plays a critical role in distinguishing the functional ranges of the EI family.

When PEP binds to the C-terminal domain of EI, an open-to-closed rearrangement is stimulated, activating the heterodimer for its autophosphorylation reaction. Relaxation dispersion NMR studies of the temperature dependence of the eEIC and tEIC open-to-closed transitions provided thermodynamic insight into the conformational change and exchange kinetics between active populations with distinct chemical shifts (Burns et al., 2022). Motions of thermophilic tEIC investigated via ^15^NH and ^13^CH_3_-ILV methyl relaxation dispersion over a temperature range of 298–343 K were predominantly localized to residues within and surrounding the active site loops, evidenced by substantial *R*_*ex*_ values. Interestingly, flexibility of eEIC were propagated from the active site to the central beta barrel of the enzyme with kinetics of chemical exchange (k_ex_) ∼4-fold faster than those of tEIC, though both enzymes undergo a concerted open-to-closed motion. In contrast, the temperature dependence of the minor state population (*P*_B_) was remarkably similar ion both enzymes, determined with the Eyring Equation over a temperature range of 298–313 K for eEIC and 313–343 K for tEIC.

A very elegant dissection of the factors conferring the thermodynamic and kinetic properties of eEIC and tEIC leveraged hybrid meso-thermophilic EIs. Proteins with active site loops from mesophilic EIC with the structural scaffold of the thermophilic EIC (etEIC) and those with thermophilic EIC active sites and mesophilic scaffolds (teEIC) required 21-point mutations each and the condition that the folded state of the enzyme remained unperturbed (Dotas et al., 2020; Burns et al., 2022). Remarkably, the hybridization of the tEIC scaffold with the mesophilic active site was sufficient to increase the rate of active site chemical exchange to levels similar to the overall mesophilic enzyme (∼4-fold increase in k_ex_). Reciprocally, the addition of the tEIC active site loop to the mesophilic scaffold increases the activation barrier for the open-to-close transition marked by an attenuated local k_ex_. Thermal denaturation curves of these enzymes produced unfolding *T*_m_ values for eEIC, teEIC, etEIC, and tEIC of 327, 300, 353, and 364 K, respectively, indicating that hybridization of mesophilic and thermophilic EIC induce the expected effect, where teEIC raises the mesophilic *T*_m_ and etEIC lowers the thermophilic *T*_m_, despite only substituting a relatively small portion of the enzymes.

Although it is not the traditional catalytic domain of EI, the C-terminal subunit plays an important allosteric role in the activation of PEP for catalysis and can catalyze PEP hydrolysis in the absence of EIN at a much slower rate, but via a similar mechanism to full-length EI. The EIC therefore presents a unique opportunity to assess PEP hydrolysis over a wide temperature range (278–343 K) in real-time via NMR. Though hydrolysis of PEP by meso-thermophilic EIC enzymes and hybrids followed Michaelis-Menten kinetics, the hybrids bound PEP less tightly than either wild-type protein based on *K*_M_ values of 300 and 500 μM for eEIC and tEIC versus a 400–1,300 μM range for etEIC and teEIC. The temperature dependence of *k*_cat_ revealed higher PEP hydrolysis rates in eEIC (meso) than tEIC (thermo) at lower temperatures, indicating that eEIC is better suited for activating PEP for the phosphoryl transfer reaction (Burns et al., 2022). Substrate specificity tests of mesophilic and thermophilic EIC and its hybrids showed that PEP analogs were more promiscuously recognized by the thermophilic EIC due to the inability of mesophilic EIC to complete the open-to-close conformational change, but that the enzyme scaffold, not the active site loop, drives promiscuous binding. Collectively, investigations of protein conformational dynamics in meso-thermophilic proteins and chimeric systems can offer novel insight into the effects of ligand specificity and overall enzymatic activity across temperatures.

## Conclusion

In this review, we provided an overview of relevant methods, theoretical considerations, tools, and case studies for investigating the temperature-dependent function of mesophilic-thermophilic enzyme pairs and the allosteric regulation of enzymes across a wide temperature range. The modulation of temperature in solution NMR, X-ray crystallography, and calorimetric experiments, as well as MD simulations, can alter the protein conformational equilibrium and biologically relevant ligand binding configurations that remain elusive at temperatures distant from those of optimal activity. Exploring such thermodynamic and structural principles are crucial for expanding our understanding of the importance of temperature modulation and adaptation in protein function, particularly in thermophilic organisms that are often studied at room temperature in the laboratory. The multi-faceted nature of protein dynamics and interplay with observable structural changes makes studies of temperature-dependent allostery highly complex. Indeed, temperature can enhance, suppress, or have no effect on allosteric coupling, and such outcomes cannot be universally ascribed to or predicted by a certain sequence, structure, intrinsic dynamic profile, or mechanism. This is further underscored by a lack of broadly defined biophysical principles that distinguish mesophilic from thermophilic proteins. Continued research in this field is essential to better understanding of how laboratory and native environmental temperatures alter chemical crosstalk within proteins.
